# T cell apoptosis characterizes severe Covid-19 disease

**DOI:** 10.1038/s41418-022-00936-x

**Published:** 2022-01-22

**Authors:** Sonia André, Morgane Picard, Renaud Cezar, Florence Roux-Dalvai, Aurélie Alleaume-Butaux, Calaiselvy Soundaramourty, André Santa Cruz, Ana Mendes-Frias, Clarisse Gotti, Mickaël Leclercq, Alexandre Nicolas, Alexandra Tauzin, Alexandre Carvalho, Carlos Capela, Jorge Pedrosa, António Gil Castro, Lucy Kundura, Paul Loubet, Albert Sotto, Laurent Muller, Jean-Yves Lefrant, Claire Roger, Pierre-Géraud Claret, Sandra Duvnjak, Tu-Anh Tran, Gina Racine, Ouafa Zghidi-Abouzid, Pierre Nioche, Ricardo Silvestre, Arnaud Droit, Fabrizio Mammano, Pierre Corbeau, Jérôme Estaquier

**Affiliations:** 1 INSERM-U1124, Université Paris, Paris, France; 2 Laboratoire d’Immunologie, CHU de Nîmes, Nîmes, France; 3 Proteomics platform, CHU de Québec - Université Laval Research Center, Québec City, Québec, Canada; 4 Computational Biology Laboratory, CHU de Québec - Université Laval Research Center, Québec City, Québec, Canada; 5 Structural and Molecular Analysis Platform, BioMedTech Facilities INSERM US36-CNRS UMS2009, Université Paris, Paris, France; 6 Life and Health Sciences Research Institute (ICVS), School of Health Sciences, University of Minho, Braga, Portugal; 7 ICVS/3B’s – PT Government Associate Laboratory, Braga/Guimarães, Portugal; 8 Department of Internal Medicine, , Hospital of Braga, Braga, Portugal; 9 Institut de Génétique Humaine UMR9002 CNRS-Université de Montpellier, Montpellier, France; 10 Service des Maladies Infectieuses et Tropicales, CHU de Nîmes, Nîmes, France; 11 Service de Réanimation Chirugicale, CHU de Nîmes, Nîmes, France; 12 Urgences Médico-Chirugicales Hospitalisation, CHU de Nîmes, Nîmes, France; 13 Service de Gérontologie et Prévention du Vieillissement, CHU de Nîmes, Nîmes, France; 14 Service de Pédiatrie, CHU de Nîmes, Nîmes, France; 15 CHU de Québec - Université Laval Research Center, Québec City, Québec, Canada

**Keywords:** Immune cell death, Microbiology

## Abstract

Severe SARS-CoV-2 infections are characterized by lymphopenia, but the mechanisms involved are still elusive. Based on our knowledge of HIV pathophysiology, we hypothesized that SARS-CoV-2 infection-mediated lymphopenia could also be related to T cell apoptosis. By comparing intensive care unit (ICU) and non-ICU COVID-19 patients with age-matched healthy donors, we found a strong positive correlation between plasma levels of soluble FasL (sFasL) and T cell surface expression of Fas/CD95 with the propensity of T cells to die and CD4 T cell counts. Plasma levels of sFasL and T cell death are correlated with CXCL10 which is part of the signature of 4 biomarkers of disease severity (ROC, 0.98). We also found that members of the Bcl-2 family had modulated in the T cells of COVID-19 patients. More importantly, we demonstrated that the pan-caspase inhibitor, Q-VD, prevents T cell death by apoptosis and enhances Th1 transcripts. Altogether, our results are compatible with a model in which T-cell apoptosis accounts for T lymphopenia in individuals with severe COVID-19. Therefore, a strategy aimed at blocking caspase activation could be beneficial for preventing immunodeficiency in COVID-19 patients.

## Introduction

Since December 2019, a new infectious respiratory disease emerged in Wuhan, China, named coronavirus disease 19 (COVID-19) caused by the severe acute respiratory syndrome coronavirus 2 (SARS-CoV-2) [1]. Patients with SARS-CoV-2 infection have reportedly had mild to severe respiratory illness. The most common symptoms on admission are fever and a cough, followed by sputum production and fatigue. Infected patients may develop pneumonia which, in severe cases, leads to fatal acute respiratory distress syndrome (ARDS). Among the determinants of disease severity, the age and gender of individuals have been proposed [2]. Several inflammatory cytokines like IL-6, or chemokines like interferon-inducible protein 10/IP-10 (CXCL10) and impaired interferon response have been linked to a poor prognosis [3–7].

A hallmark of critical COVID-19 is lymphopenia, observed in up to 63% of COVID-19 patients [8]. In addition, a defect in Th1 cell immunity [9–12] and T cell depletion in lymphoid tissues [13] have been associated with severe disease outcome in COVID-19 patients. In the context of individuals infected by the human immunodeficiency virus (HIV), a defect in T helper cell (Th) 1 immunity and T cell depletion have been associated with abnormal priming of T cells, causing them to die by apoptosis [14–17]. Apoptotic cell death involves both the extrinsic (death receptors such as CD95/Fas or TRAIL) and intrinsic pathways (members of the Bcl-2 family) involving cysteine proteases, namely caspases [18], in which caspase-3 causes apoptosis. Although CD95 engagement by its counterpart FasL has a minor impact on T cells from healthy donors, Th1 cells and activated T cells are more prone to dying through Fas/FasL interaction [19–23]. In addition, T cells from HIV-infected individuals are highly sensitive to FasL-mediated cell death in which caspase activation is critical. Thus, blocking caspase activation prevents T cell death [24–28].

A recent report has indicated an increase in Fas/CD95 expression in T cells [29] but, so far, it is not known whether lymphoid T cells are more likely to die by apoptosis during SARS-CoV-2 infection. Recent findings suggest that syncytia formation in the lung may contribute to the depletion of T lymphocytes during SARS-CoV-2 infection [30]. However, several programmed cell death pathways, pyroptosis, necroptosis and PANoptosis, have also been described [31–35]. Thus, active NRLP3 inflammasome has been observed in the myeloid cells of COVID-19 patients [36], and a combination of TNF-α and IFN-γ-mediated macrophage PANoptosis [37].

The aim of this study was to analyze the levels of sFasL and CD95/Fas relative to the propensity of T cells to die in SARS-CoV-2 infected individuals. Herein, we found a positive correlation between plasma levels of sFasL, CD95/Fas expression on T cells and T cell apoptosis in COVID-19 patients. Furthermore, the expression of Bcl-2 family members are modulated in T cells. These were correlated with the extent of lymphopenia and CXCL10 associated with disease severity in COVID-19 patients. We demonstrated that Q-VD, a broad caspase-inhibitor, could prevent T cell apoptosis and enhance Th1 mRNA expression, opening up new horizons for the treatment of COVID-19.

## Results

### 1) Increased levels of plasma sFasL and Fas expression on T cells in SARS-CoV-2 infected individuals

**Fig. 1:**
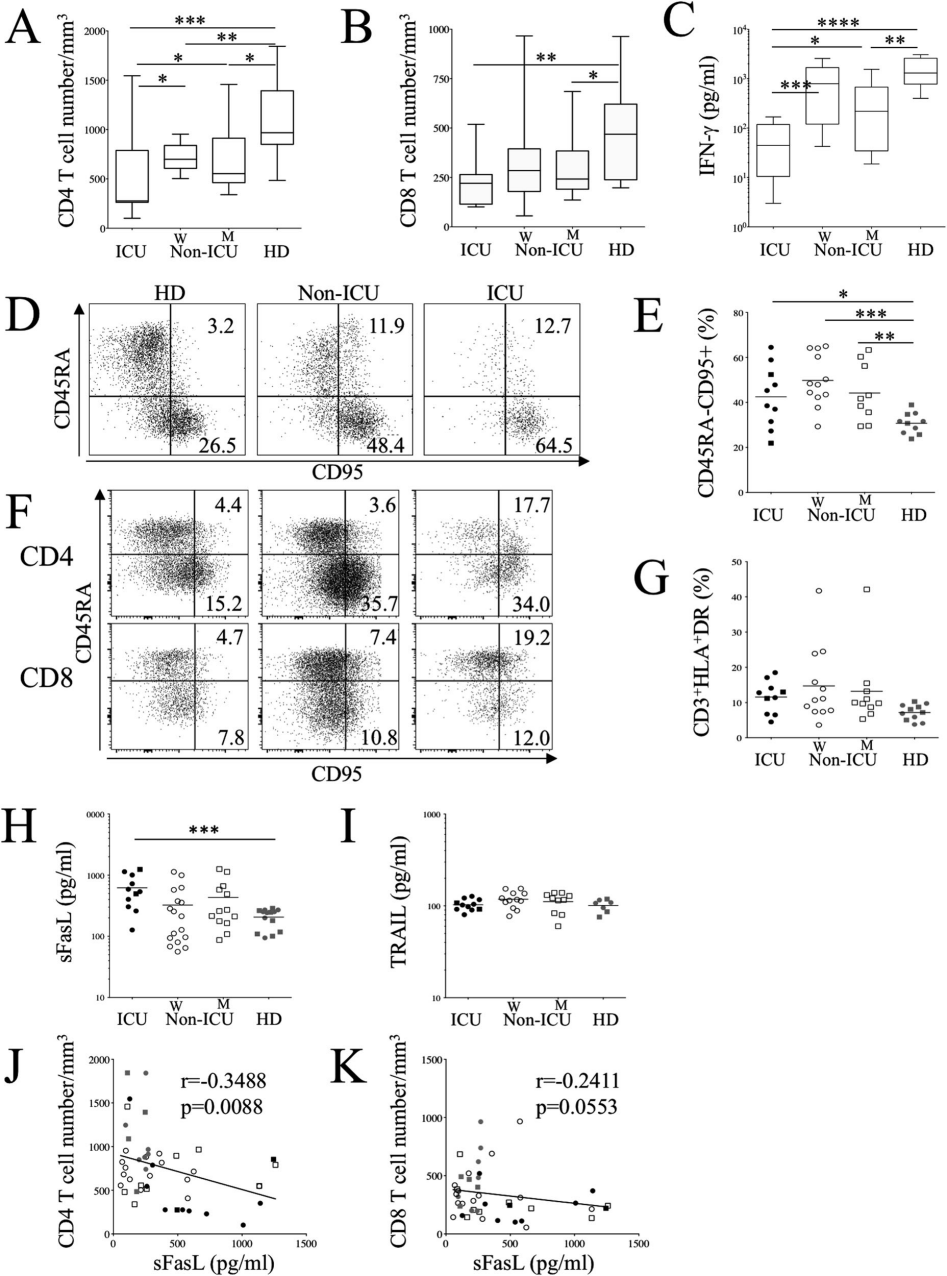
CD95 and soluble FasL correlate with lymphopenia in COVID 19 patients. **A**, **B** CD4 and CD8 T cell numbers in ICU, non-ICU patients and HDs. **C** PBMCs were stimulated with anti-CD3 and anti-CD28 mAbs for 48 h and IFN-γ was quantified in culture supernatants. **D** Dot-plots showing CD95 expression on memory (CD45RA^-^) T cells assessed by flow cytometry. **E** Percentages of memory T cells expressing CD95. **F** Dot-plots showing CD95 expression on memory (CD45RA^-^) CD4 and CD8 T cells. **G** Percentages of activated T cells (HLA-DR expression). **H**, **I** Plasma levels of sFasL and TRAIL in the different groups of patients. Each dot represents one individual. Marker shape represents gender (round: women; square: men). Statistical analysis was performed using a Mann–Whitney *U* test. *p < 0.05, **p < 0.01, and ***p < 0.001). **J**, **K** Correlation between CD4 and CD8 T cell numbers and sFasL. Values of Spearman correlation are shown in the panels.

Intensive care unit patients (namely ICU) and non-ICU patients were included in our study (Table 1). All patients included in our study were hospitalized from April to July 2020. We observed a significant decrease in CD4 T cell counts in ICU compared to HDs (501 ± 126/mm^3^ versus 1114 ± 131/mm^3^, *p* = 0.0009; Fig. 1A) as well as in CD8 T cell counts (232 ± 38/mm^3^ versus 465 ± 71/mm^3^, *p* = 0.006; Fig. 1B). We observed a gradual decline in the secretion of Th1 cytokine IFN-γ in relation to the status of COVID-19 patients and consistent with previous reports [9, 10] (Fig. 1C). We first analyzed the expression of CD95 on T cells. Our results indicated higher expressions of CD95/Fas on memory (CD45RA^neg^) T cells (Fig. 1D, E), including both CD4 and CD8 T cells (Fig. 1F), which was not related to higher immune activation in COVID-19 patients as compared to HDs (Fig. 1G). Interestingly, both ICU and non-ICU demonstrated higher levels of CD95/Fas expression as compared to HDs (Fig. 1E), and these levels were positively correlated with CD4 T cell numbers (*p* = 0.019). However, B cells (CD20) did not express higher levels of CD95 (Fig. S1A, B). We also found that CD4 T cells in ICU also expressed higher levels of exhaustion markers, TIM-3 and LAG-3, compared to HDs [38, 39], whereas only LAG-3 was significantly higher in CD8 T cells (supplemental Fig. 2A). In contrast, we did not observe any difference in the expression of CXCR4, CCR5 and CX3CR1 (Fig. S2B), previously reported to contribute to T cell redistribution [40].

**Table 1:** Clinical characterization of the transversal COVID-19 analysis.

Age, years			
Mean (SD)	57.8 (21)	67.5 (21)	75.9 (15)
Range	28–95	29–96	43–95
Gender, *n* (%)			
Female	7 (50)	16 (53)	9 (82)
Male	7 (50)	14 (47)	2 (8)
Underlying diseases, *n* (%)			
Diabetes		7 (23)	2 (18)
Cancer		4 (13)	2 (18)
Autoimmune disease		2 (7)	1 (9)
Symptoms, days			
Mean(SD)	none	6.2 (9)	12.3 (10)
Symptomatology*			
1- Deterioration of general condition		12/30	
2- Dyspnea		18/30	
3- ARDS		0/30	11/11
Treatment, *n* (%)			
Lopinavir and Ritonavir		5/30 (17)	3/11 (27)
Prednisolone	none	3/30 (10)	3/11 (27)
Dexamethasone		2/30 (0.7)	1/11 (0.9)
Antibiotics		1/30 (0.3)	8/11 (73)

^*^Clinical Score of 1–2 is defined as non-intensive care unit (non-ICU) individuals, and three as intensive care unit (ICU) patients. Four ICU patients died during hospitalization.

Having observed a positive correlation between Fas/CD95 and lymphopenia, we then quantified plasma levels of sFasL as well as those of the TNF-related apoptosis-inducing ligand (TRAIL). We demonstrated that plasma concentrations of sFasL, but not TRAIL, increased more in ICU than in HDs (621 ± 111 pg/ml versus 206 ± 19 pg/ml, *p* = 0.0002) (Fig. 1H and I) and were high in half of the non-ICU (above 350 pg/ml, Fig. 1H). Our results indicated a negative correlation between CD4 T cell numbers and concentrations of sFasL (*p* = 0.0088), whereas only a trend was observed for CD8 T cell numbers (*p* = 0.05). A similar negative correlation was observed between CD95/Fas and CD4 T cell numbers (*p* = 0.03) (Fig. 1J, K).

Thus, our results demonstrate a high level of plasma sFasL and CD95/Fas in COVID-19 patients, both of which are associated with T cell lymphopenia.

### 2) T cell death in SARS-CoV-2 infected individuals

**Fig. 2:**
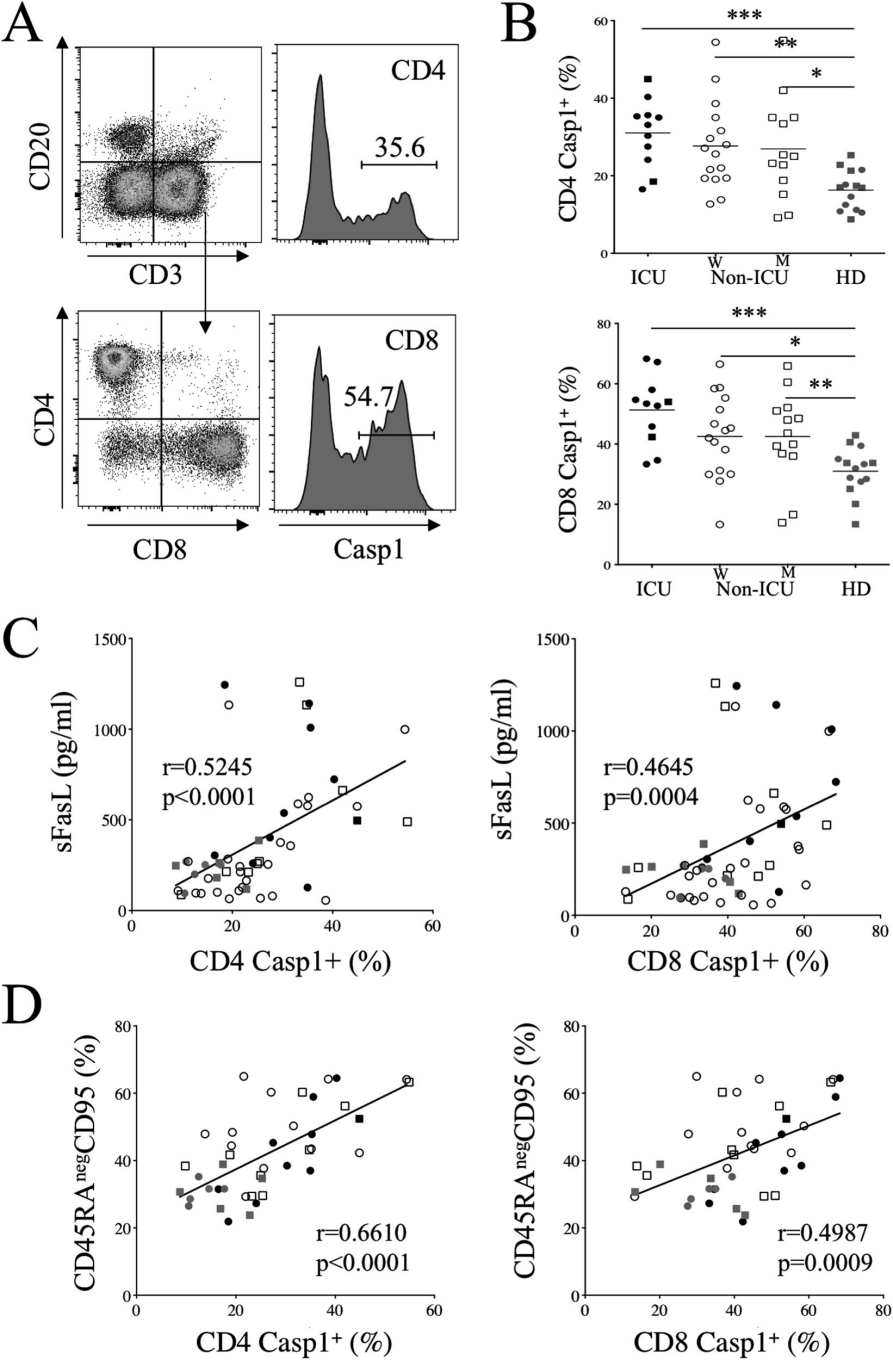
Caspase activation in T cells correlates with CD95 and soluble FasL in COVID 19 patients. **A** Caspase activity of CD4 and CD8 T cells was quantified by flow cytometry using fluorescent caspase-1 substrate. **B** Percentages of CD4 and CD8 T cells expressing fluorescent caspase substrates are shown. Each dot represents one individual. Marker shape represents sex gender (rounded: women; squared: men). Statistical analysis was performed using a Mann–Whitney *U* test. *p < 0.05, **p < 0.01, and ***p < 0.001). **C** Correlation between sFasL and caspase-1 activation in CD4 and CD8 T cells. **D** Correlation between the percentages of CD45RA^-^ T cells expressing CD95 and caspase-1 activation in CD4 and CD8 T cells. Values of Spearman correlation are shown in the panels.

Since caspases are activated during T cell death [25, 27, 28, 41], we used fluorescent caspase substrates. COVID-19 patients’ T cells expressed higher caspase activity than those of HDs (Fig. 2B). The percentages of T cells expressing caspase-1 activity were significantly higher in ICU (CD4, 31.1 ± 2.6%; CD8, 51.3 ± 3.5%) as well in non-ICU when compared to HDs (CD4, 16.3 ± 1.4%; CD8, 31 ± 2.1%; Fig. 2B). We also observed higher levels of caspase-3 activity both in CD4 and CD8 T cells from ICU and non-ICU compared to HD (Figs. S3A, B). Furthermore, we observed strong positive correlations between caspase-1 activity in CD4 and CD8 T cells on the one hand, and plasma sFasL levels (*p* < 0.0001 and *p* = 0.0004, respectively, Fig. 2C) as well as with the frequency of memory T cells expressing CD95/Fas (*p* < 0.0001 and *p* = 0.0009, respectively, Fig. 2D). Similarly, the percentages of T cells expressing caspase-3 activity were positively correlated with plasma levels of sFasL (Fig. S3C) and the frequency of memory CD95/Fas T cells (Fig. S3D).

**Fig. 3:**
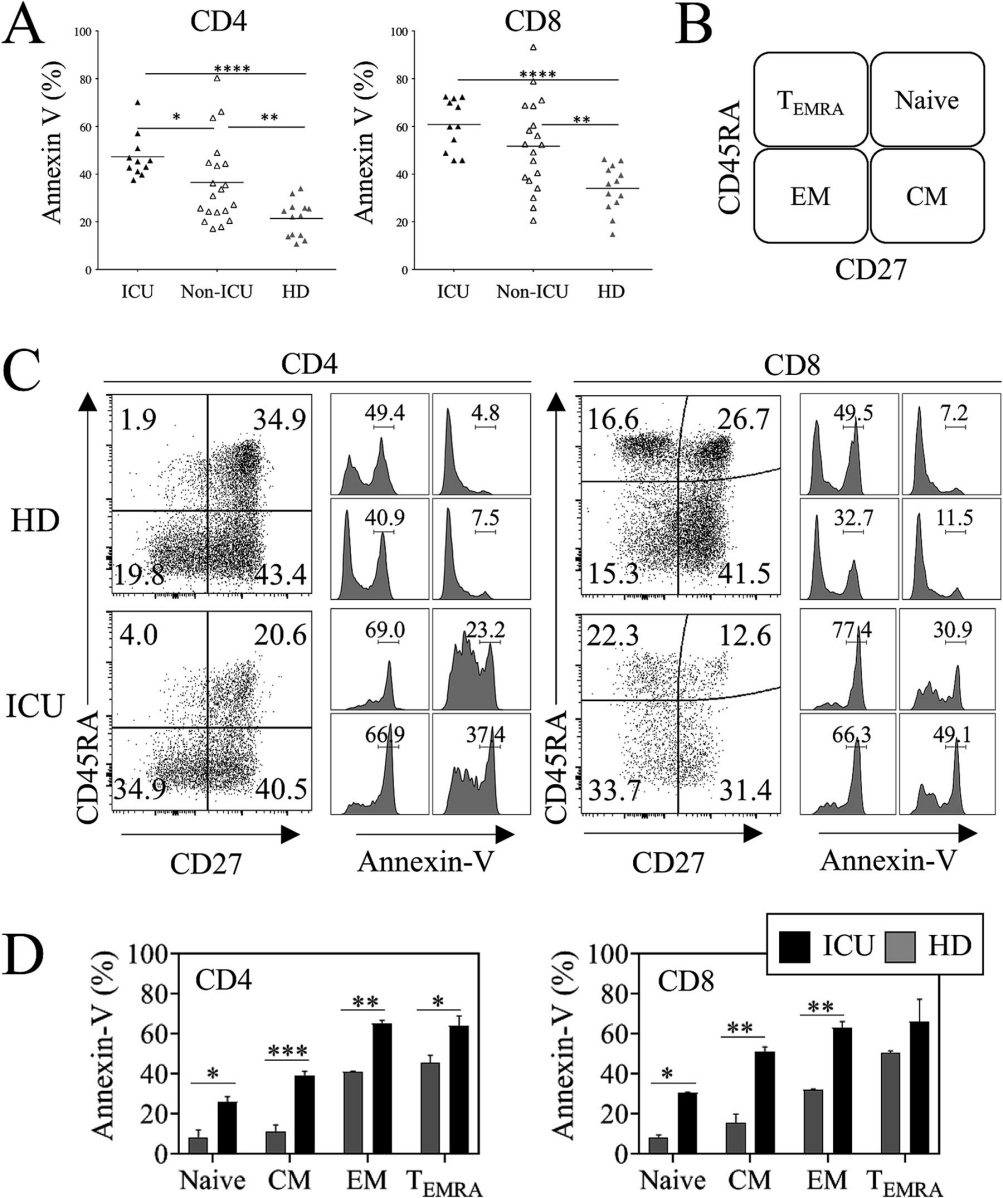
Memory CD4 and CD8 T cells are more prone to dying by apoptosis in COVID 19 patients. **A** Percentages of CD4^+^ and CD8^+^ T cells expressing annexin V. Phosphatidyl serine exposure was assessed by flow cytometry in ICU, non-ICU patients and HDs. Each dot represents one individual. **B** Subsets of CD4 and CD8 T cells were defined using CD45RA and CD27. **C** Representative staining of annexin V in the different subsets of CD4 and CD8 T cells. **D** Histograms show the means ± SEM of CD4 and CD8 T cell subsets from four HDs and four ICU patients. Statistical analysis was performed using a Mann–Whitney *U* test (*p < 0.05, **p < 0.01, ***p < 0.001, and ****p < 0.0001).

We then analyzed phosphatidyl serine (PS) exposure, which is externalized during T cell death. Our data indicated that both CD4 (47.2 ± 2.8%, *p* < 0.0001) and CD8 T cells (60.8 ± 3.2%, *p* < 0.0001) from ICU demonstrate higher PS-exposure in comparison to T cells from HDs (21.4 ± 2.1% and 34.1.8 ± 2.7%, respectively) (Fig. 3A). A significant increase in the percentages of annexin V-labeled T cells was also already obvious in non-ICU (CD4, 36.5 ± 3.9% and CD8, 51.7 ± 4.3%) when compared to HDs (Fig. 3A).

We then assessed the nature of T cell subsets, which were primed to die. T cells can be subdivided into distinct populations: naïve (CD27+CD45RA+), central memory (CM, CD27+CD45RA^−^), effector memory (EM, CD27^-^CD45RA^−^) and terminal differentiated (T_EMRA_, CD27^−^CD45RA+) (Fig. 3B). We found that EM and T_EMRA_ T cell subsets expressed significantly higher levels of annexin V than the naïve and CM T cell subsets (Fig. 3C, D).

**Fig. 4:**
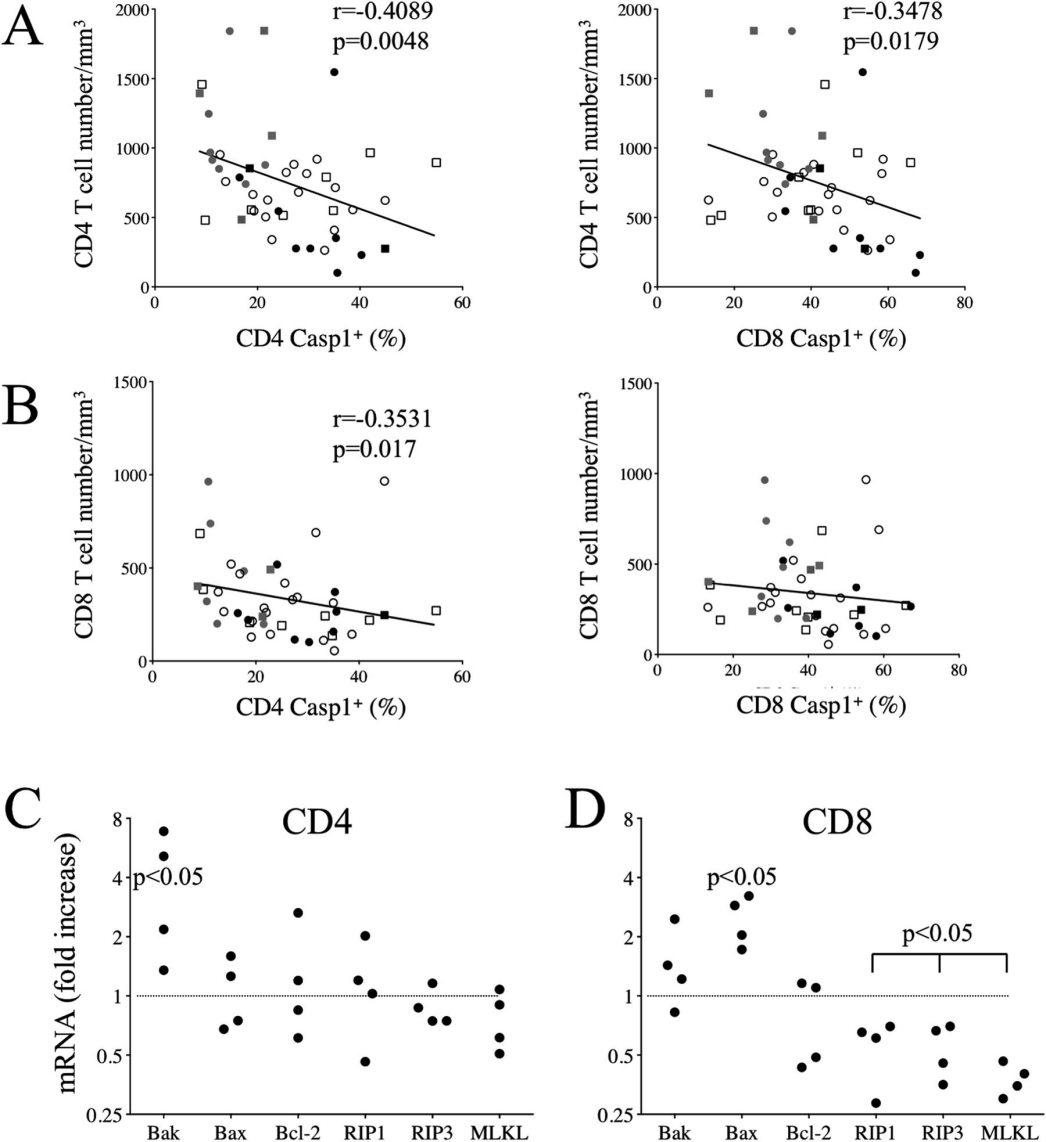
Lymphopenia correlates with caspase activation in COVID-19 patients. **A** Correlation between CD4 T cell numbers and caspase activity in CD4 and CD8 T cells. **B** Correlation between CD8 T cell numbers and caspase activity in CD4 and CD8 T cells. Values of Spearman correlation are shown in the panels. **C**, **D** Expression of mRNA coding for Bax, Bak, Bcl-2, RIP1, RIP3 and MLKL genes in purified CD4 and CD8 T cells. Each dot represents a pool of three COVID-19 individuals due to the limited amounts of cells. Results are expressed as fold increase in comparison with the mean values of three HD pools as shown by the dotted line. Statistical analysis was performed using a paired Mann–Whitney *U* test.

As lymphopenia characterizes COVID-19 patients, we plotted both T cell numbers against the percentages of T cells expressing caspase activity. Our results indicated a negative correlation between CD4 T cell counts and caspase activity in both CD4 and CD8 T cells (Fig. 4A, *r* = −0.4089, *p* = 0.0048 and Fig. 4B, *r* = −0.3478, *p* = 0.0179, respectively). By contrast, no correlation was observed between the caspase activity of CD8 T cells and CD8 T cell counts (Fig. 4B). This result may suggest that abnormal apoptosis of CD8 T cells could be the consequence of CD4 T cell death in COVID-19, shortening CD8 T cell survival (CD8 helpless) [42, 43].

We then analyzed the expression of Bax, Bak and Bcl-2 transcripts in purified CD4 and CD8 T cells. Compared to HDs, we found higher levels of Bak and Bax transcripts, in CD4 and CD8 T cells from COVID-19 individuals, respectively (Fig. 4C, D). Our results also indicated a lower expression of Bcl-2 transcripts only in CD8 T cells. Similar profiles were reported in T cells during Aids [44, 45]. We extended the analyses of RIP1, RIP3 and MLKL mRNA that contribute to necroptosis/PANaptosis [35]. Although no significant difference was observed in CD4 T cells, these transcripts were lower in CD8 T cells from COVID-19 individuals compared to HDs (Fig. 4C, D).

Altogether, these data demonstrated that caspase activation and T cell apoptosis are associated with T cell lymphopenia in COVID-19 individuals.

### 3) T cell apoptosis in SARS-CoV-2 infected individuals correlates positively with CXCL10

**Fig. 5:**
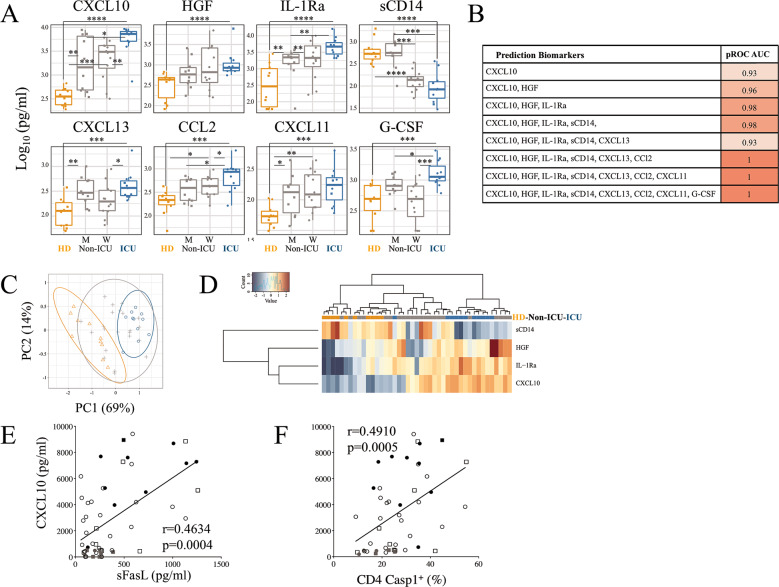
Correlation between CXCL10 and caspase activity in COVID 19 patients. **A** Boxplots of the eight most informative cytokine biomarkers ranked by information gain using Biocomb R package. *Y*-axis shows cytokine concentration (pg/mL). Colors represent patient groups (orange: HDs; gray: non-ICU; blue: ICU) and marker shape represents gender (square: men Boxplot2; round: women Boxplot3). Significant variations between groups based on Student’s *t*-test are showed above each graph (*p < 0.05; **p < 0.01; ***p < 0.001; ****p < 0.0001). **B** AUCs of pROC were computed using Meval R package by adding genes, ranked by information gain using Biocomb R package, to a naïve Bayes model computed with the R caret Package. **C** Principal Component Analysis performed with R software based on a short cytokine signature (CXCL10, HGF, IL-1Ra and sCD14). Ellipses were calculated by R and correspond to a 95% interval. **D** Heatmap on cytokine concentration values for each sample with Ward’s hierarchical clustering in rows (cytokine) and columns (patients – HDs (orange); non-ICU (gray); ICU (blue)). **E**, **F** Correlation between levels of CXCL10 and plasma sFasL and between caspase activity in CD4 T cells. Values of Spearman correlation are shown in the panels.

COVID-19 is characterized by inflammation, in which the expression of CXCL10 correlates with disease severity [6, 46]. We evaluated 32 soluble factors in the plasma of COVID-19 patients. We found that plasma levels of CXCL10, IL-1Ra, hepatocyte growth factor (HGF), and soluble CD14 (sCD14,) are biomarkers that discriminate ICU from HDs (Fig. 5A–D). The AUC (Area Under the ROC Curve) was 0.98 based on these four biomarkers and associated with disease severity (Fig. 5B). Thus, CXCL10 is higher in ICU compared to HDs (ICU, 6523 ± 777 pg/ml versus HDs, 373 ± 49 pg/ml, *p* < 0.0001) as well as CXCL11 (ICU, 206 ± 49 pg/ml versus HDs, 59 ± 8 pg/ml, *p* = 0.0002) and CXCL13 (ICU, 522 ± 162 pg/ml versus HDs, 143 ± 32 pg/ml, *p* = 0.0003) (Fig. 5A). The dynamics of these C–X–C chemokines in COVID-19 during hospitalization (*n* = 18 COVID-19 patients, Table 2) showed an early increase from day 5 to day 15 after hospitalization, and their levels declined thereafter (Fig. S3). By contrast, among the C–C chemokines tested, CCL2 increased the most in the plasma of ICU compared to HDs (848 ± 185 pg/ml versus 221 ± 33 pg/ml, *p* = 0.0006; Fig. 5A). IL-1Ra, an inhibitor of inflammation [47] was elevated in ICU (ICU, 5630 ± 1275 pg/ml versus HDs, 733 ± 288 pg/ml, *p* < 0.0001; Fig. 5A) as well IL-6 (ICU, 85.6 ± 22.5 pg/ml versus HDs, 27.4 ± 9.6 pg/ml, *p* = 0.019 Fig. S4). It should be noted that sCD14, considered as a biomarker of disease severity and comorbidities in HIV-infected individuals [48] when it increases, actually decreased in COVID-19 patients (ICU, 122 ± 33 pg/ml versus HDs, 723 ± 175 pg/ml, *p* < 0.0001; Fig. 5A). HGF concentration was also higher in ICU than in HDs (1516 ± 626 pg/ml versus 381 ± 67 pg/ml, *p* < 0.0001, Fig. 5A). This could be of importance as it has been previously reported that stimulation of the hypoxia inducible factor-1 by HGF was associated with pulmonary arterial hypertension [49]. Among the three colony-stimulating factors (M-CSF, G-CSF and GM-CSF), G-CSF was higher in ICU (ICU, 1417 ± 219 pg/ml versus HDs, 552 ± 97 pg/ml, *p* = 0.0003, Fig. 5A and Fig. S4). This is of interest as it had previously been reported that intrapulmonary infections raised G-CSF production in the lungs [50]. Furthermore, IL-18, which is cleaved by caspase-1 [32, 33], was higher in ICU than in HDs (ICU, 1137 ± 278 pg/ml versus HDs, 639 ± 30 pg/ml, *p* = 0.015) (Fig. S5). Thus, the Principal Component Analysis (PCA) (Fig. 5C) as well the heat map presented in Fig. 5D demonstrated the ability of CXCL10, IL-1Ra, HGF, and sCD14 to classify COVID-19 patients.

**Table 2:** Clinical characterization of the longitudinal COVID-19 analysis.

Parameter	*N* or mean (% or range)
Gender, *n* (%)	
Female	9 (50)
Male	9 (50)
Age, years (range)	72 (44–92)
Underlying diseases, *n* (%)	
Autoimmune disease	1 (6)
Cancer history	3 (17)
Hypertension	10 (56)
Diabetes	3 (17)
Chronic obstructive pulmonary disease	3 (17)
Other respiratory disease	1 (6)
Chronic kidney disease	2 (11)
Symptoms	
Days of symptoms before admission	8 (0-21)
Cough at admission	11 (61)
Dyspnea at admission	12 (67)
Fever at admission	12 (67)
Clinical score*	
1 - Hospital admission	2 (11)
2 - Low 0_2_ supplement	9 (50)
3 - Moderate 0_2_ supplement	3 (17)
4 - High 0_2_ supplement	0 (0)
5 - Mechanical Ventilation	4 (22)
Treatment	
No medication to the infection	2 (11)
Hydroxychloroquine (monotherapy)	4 (22)
Hydroxychloroquine + azithromycin	4 (22)
Any of the above + corticosteroids	3 (17)
Corticosteroids (monotherapy)	1 (6)

^*^Clinical Score of 1–3 is defined as moderate disease and 4–5 as severe disease. Patients were classified on clinical score of 1–6 according to worst disease stage observed. Two patients died during hospitalization (Score 5).

We then plotted the levels of CXCL10 against plasma levels of sFasL and caspase activity in T cells. We noticed a strong correlation between CXCL10 with sFasL (*p* = 0.0004, Fig. 5E) and with caspase activation in CD4 T cells (*p* = 0.0005, Fig. 5F).

We thus demonstrated that levels of plasma sFasL and T cell death correlate with the level of CXCL10, a marker of COVID-19 severity.

### 4) Prevention of T cell death by caspase inhibitors

**Fig. 6:**
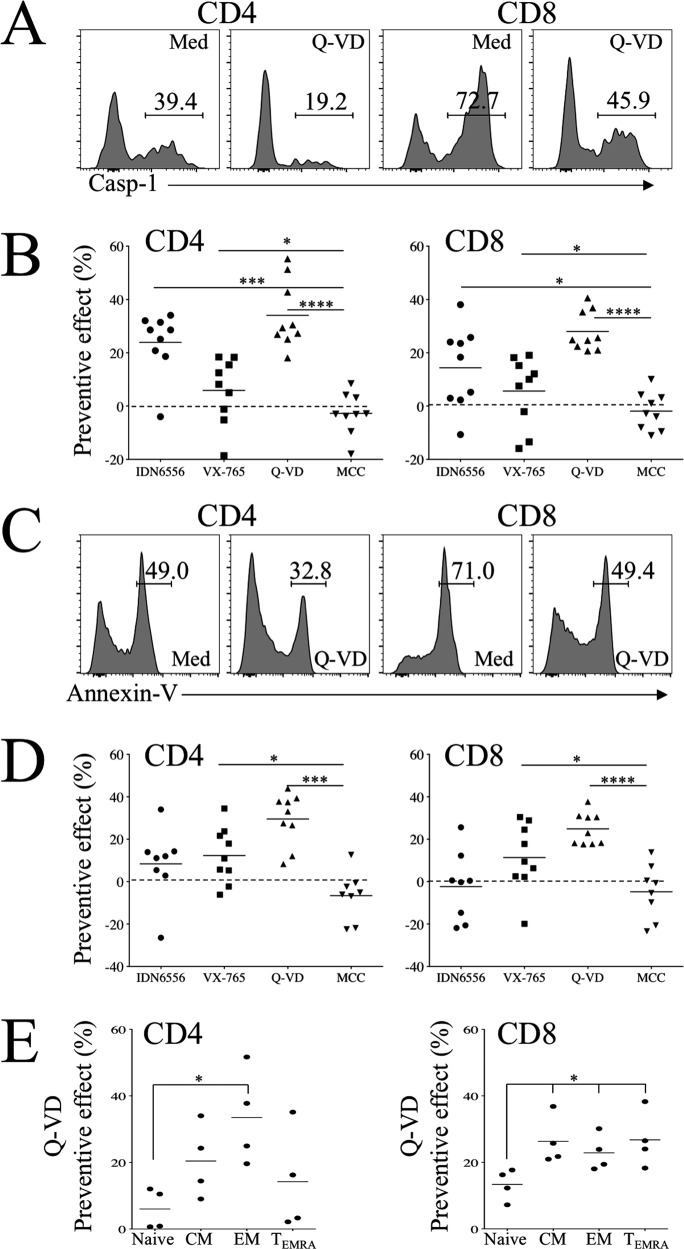
Inhibition of caspase prevents T cell apoptosis. **A** Flow cytometry of CD4 and CD8 T cells expressing caspase activity in the absence (Med) or presence of Q-VD-Oph. **B** Inhibition of caspase activity (preventive effect) in the presence of IDN6556, VX-765, Q-VD and MCC950. Percentages were calculated as follows: (Med-Inh/Med)*100. Each dot represents one individual. **C** Flow cytometry of CD4 and CD8 T cells expressing annexin V in the absence (Med) or presence of Q-VD. **D** Inhibition of apoptosis in the presence of IDN6556, VX-765, Q-VD and MCC950. Percentages were calculated as follows: (Med-Inh/Med)*100. **E** Inhibition of T cell subsets to undergo death in the presence of Q-VD. Each dot represents one individual. Statistical analysis was performed using a Mann–Whitney *U* test (*p < 0.05, ***p < 0.001, and ****p < 0.0001).

**Fig. 7:**
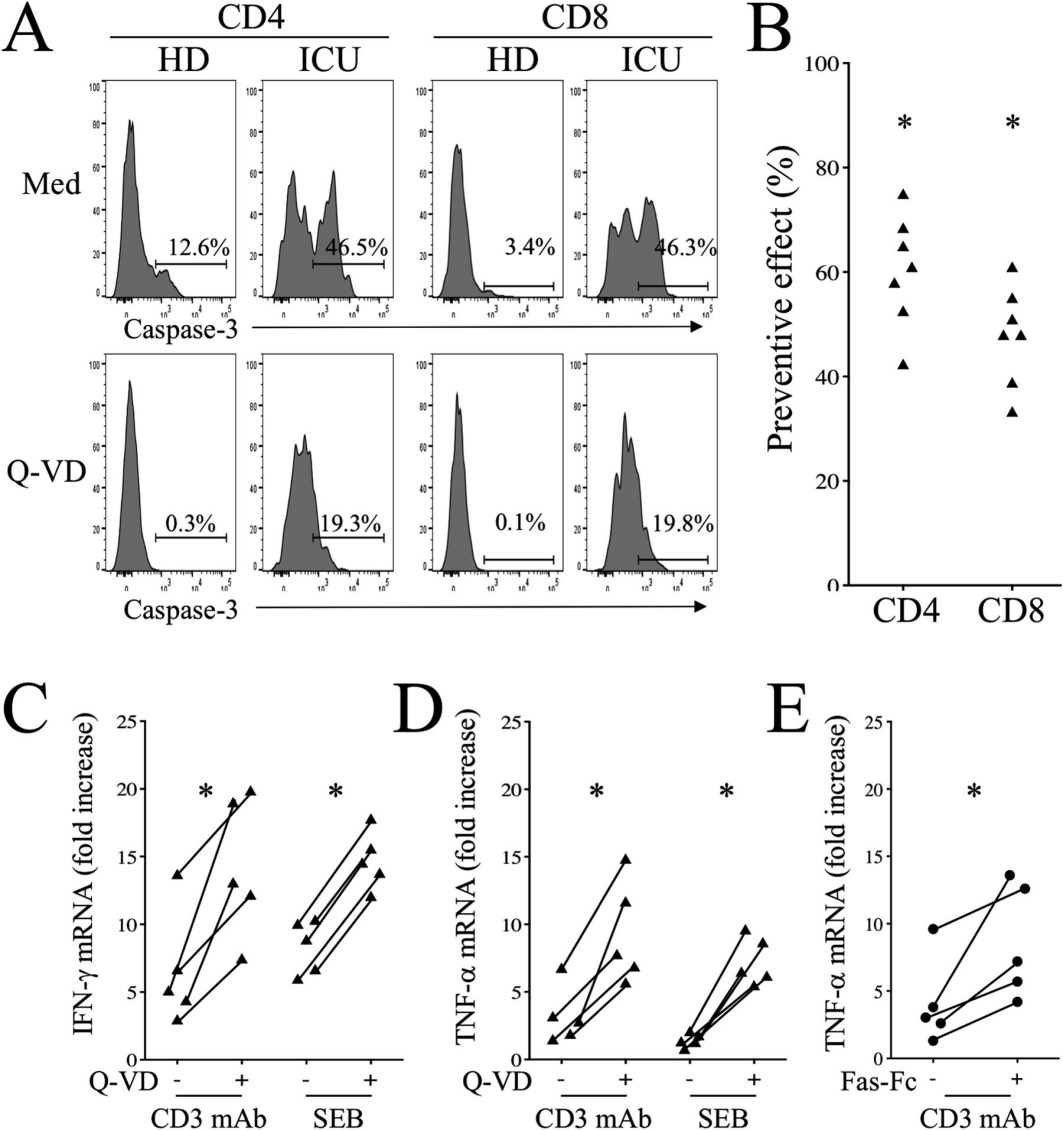
Caspase-3 inhibition enhances Th1 mRNA expression. **A** Flow cytometry of CD4 and CD8 T cells expressing active caspase-3 (Caspase-3 mAb) in the absence (Med) or presence of Q-VD. **B** Inhibition of active caspase3 (preventive effect) in the presence of Q-VD. Percentages were calculated as follows: (Med-Inh/Med)*100. Each dot represents one individual. **C**, **D** IFN-γ and TNF-α mRNA expression in cells activated with either CD3 mAbs or SEB in the absence or presence of Q-VD. Results are expressed as fold increases compared to unstimulated cells. **E** TNF-α mRNA expression in cells activated with CD3 mAbs in the absence or presence of Fas-Fc. Each dot represents one individual. Statistical analysis was performed using a paired Mann–Whitney *U* test (*p < 0.05).

**Fig. 8:**
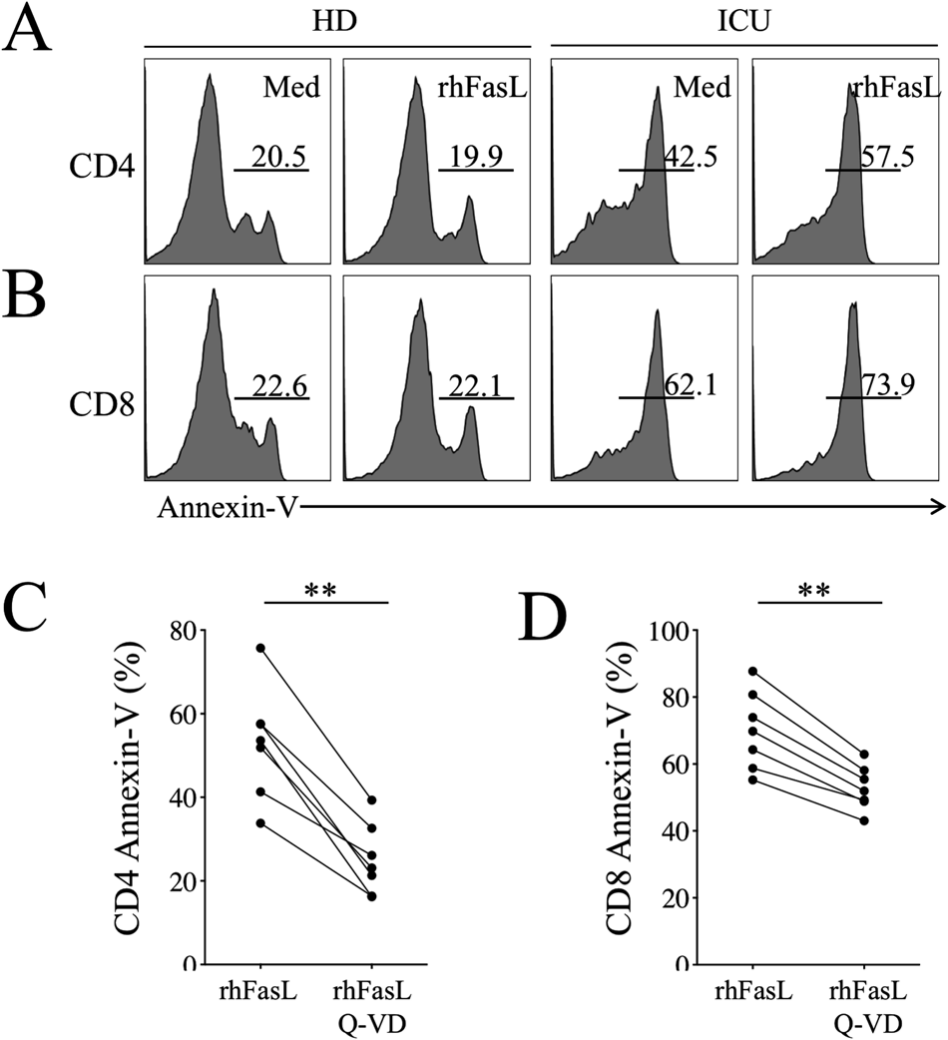
Inhibition of caspase prevents FasL-mediated T cell death. Flow cytometry of CD4 (**A**) and CD8 T cells (**B**) expressing annexin-V in the absence (Med) or presence of recombinant human FasL (rhFasL, 200 ng/ml). **C**, **D** Q-VD reduced CD4 and CD8 T cell death in the presence of rhFasL Histograms show the percentages of CD4 (**C**) and CD8 T cells (**D**) expressing annexin-V. Each dot represents one individual. Statistical analysis was performed using a paired Mann–Whitney *U* test (**p < 0.01).

The observation that T cells undergo death spontaneously after ex vivo culture without stimuli suggested that T cells had received a lethal hit in vivo in the context of SARS-CoV-2 infection, so we thought the best approach would be to inhibit caspases [18]. We assessed the impact of several specific inhibitors, namely VX-765 (Belnacasan, specific to caspase-1 and -4), IDN6556 (Emricasan, a pan-caspase inhibitor), Q-VD (a pan-caspase inhibitor, [51]), and MCC950 (an inhibitor of the NLRP3 inflammasome complex). The efficacy of these compounds was tested in either THP1 monocytic cells or primary PBMC stimulated with LPS [52–54] (Fig. S6). Our results demonstrated that Q-VD inhibited both caspase activity (Fig. 6A, B) and PS exposure (Fig. 6C, D) in CD4 and CD8 T cells from COVID-19 individuals. Q-VD behaved better than VX-765 in preventing caspase activation and PS exposure, whereas IDN6556 decreased caspase activity and had no preventive effect on PS exposure (Fig. 6B–D). By contrast, MCC950 had no preventive effect (Fig. 6B–D). Furthermore, we demonstrated that Q-VD blocks active caspase-3 in CD4 (60 ± 4% of decrease) and CD8 T cells (47.9 ± 3.5% of decrease) (Fig. 7A, B). Q-VD prevented the death of EM CD4 T cells, which are essential for cell-mediated immunity, and of memory CD8 T cells subsets, which are strongest in expressing cytotoxic effector molecules [40, 55] (Fig. 6E). Having observed that COVID-19 individuals displayed lower levels of IFN-γ, we assessed whether Q-VD restores functional T cells. Our results demonstrated higher levels of Th1 transcripts (IFN-γ and TNF-α) in activated T cells in the presence of Q-VD compared to untreated cells (Fig. 7C, D). Q-VD also prevented the death of T cells incubated in the presence of recombinant sFasL, shown to be active in inducing the death of jurkat cells (Fig. S7). Thus, CD4 and CD8 T cell apoptosis were significantly reduced by more than 52.2% ± 0.8 and 24.2% ± 0.3, respectively (Fig. 8). In contrary, sFasL had a minor impact on T cells from HDs as consistent with previous reports [19–21, 24, 25]. Furthermore, sFas-Fc, which blocks FasL-mediated jurkat cell death (Fig. S7), increased the levels of TNF-α transcripts of CD3-activated T cells from COVID-19 individuals (Fig. 7E).

As caspase inhibition may result in necroptosis [56, 57], we evaluated the synergetic effect of RIPK3 inhibitors including GSK-872 and dafrafenib. Although RIPK3 inhibitors did not prevent spontaneous T cell death in the absence of Q-VD, they had no synergetic effect with Q-VD suggesting that necroptosis had a minor role in the occurrence of T cell death (Fig. S8). This observation may be consistent with the absence of increased expression of necroptotic genes in T cells (Fig. 4C, D).

Consequently, Q-VD may represent an attractive molecule for COVID-19 patients by preventing the apoptosis of both CD4 and CD8 T cells and increasing Th1 profiles.

## Discussion

We hereby demonstrated that CD4 and CD8 T cells from COVID-19 patients are more likely to die by apoptosis, and that blocking caspase activation using Q-VD prevents T cells from dying and enhances Th1 profiles. We found higher levels of CD95 expression on T cells as well as sFasL in the plasma of COVID-19 patients, both associated with higher levels of caspase activation and PS exposure on T cells. Interestingly, transcripts of the pro-apoptotic Bcl-2 family members, Bax and Bak, are up-regulated. The propensity of CD4 T cells to die is positively correlated with T cell lymphopenia and higher levels of CXCL10, both markers of disease severity. Therefore, a strategy aiming to block caspase activation using Q-VD could be beneficial for preventing lymphopenia, improving competent immune cell survival, and preventing fatal outcomes in COVID-19 patients.

While we observed a strong effect of Q-VD in preventing caspase-3 activation, an apoptotic effector caspase, and PS exposure, we did not observe a major impact of inflammasome/pyroptosis inhibitors (VX765, IDN6556, and MCC950) nor of necroptosis inhibitors (GSK-872 and Dafrafenib), indicating that T cell death is related to apoptosis in COVID-19. Furthermore, the absence of viral infection of T cells excludes a direct role of SARS-CoV-2-mediated cell death. Interestingly, unlike T cells, myeloid cells undergo necroptosis/PANaptosis, which may amplify inflammation and disease outcome [36, 37]. Thus, despite the absence of viral infection and higher levels of immune activation (HLA-DR expression), CD4 T cells from COVID-19 patients expressed more Fas/CD95 and Bak transcripts, and are more prone to die compared to HDs. These results suggested that environmental factors or myeloid-derived suppressor cells may contribute in the dysregulation of T cells [25, 58]. Recent findings also indicate that the cellular syncytia observed in the lungs of dead patients may contribute to the elimination of T cells by a fusion mechanism [30]. Thus, multinucleated giant cells may be the hallmarks of phagocytosis of dying T cells, which, as we observed, are more likely to externalize PS residues. A similar process is responsible for the physiological PS-exposing aged and dead erythrocytes being eliminated from the circulation at lung level [59, 60].

Dysregulation of CD4 T cell apoptosis in COVID-19 patients helps to explain some recent findings. Thus, CD4 T cell apoptosis provide support for a lower Th1 cell immunity [9–12]. Herein, Q-VD, which prevents apoptosis, enhances the expression of Th1 transcripts. CD4 T cell apoptosis may also provide support for CD4 T cell depletion in lymphoid tissues and defective germinal center (GC) formations [13], which are essential for B cell and affinity antibody maturation. Several reports also indicated a lack of anti–spike IgG response in cellular non-responders to SARS-CoV-2 antigens [61] that is more pronounced in males than in females [2]. Memory B cells in seriously-affected patients harbored low mutation frequencies in their variable region genes [62], indicating suboptimal maturation consistent with defective GC formation [13]. Interestingly, during Ebola virus infection, premature T cell death due to apoptosis was associated with a lower B-cell response [63]. Therefore, the impact of T cell apoptosis in COVID-19 patients merits a thorough investigation, particularly in relationship with the development of the humoral response and long-term anti-SARS-CoV-2 immunity. Our observation that Q-VD prevents CD4 T cell apoptosis and enhances Th1 response suggests that a Q-VD-based treatment might sustain the immune response in ICU patients and help to reduce the severity of the disease and death [64].

In addition to CD4, we found that CD8 T cells are also dying in severely-affected COVID-19 patients compared to HDs, particularly memory CD8 T cell subsets, which are the most potent in expressing cytotoxic effector molecules [40, 55]. The death of CD4 T cells may contribute to the greater likelihood of CD8 T cell apoptosis, since help from CD4 is crucial for the development of memory and effector CD8 T cells, given that helpless CD8 T cells are exhausted and shorter-lived cells [42, 65]. CD8 T cells expressed higher levels of Bax and lower levels of Bcl-2, which are key regulators of cell death. Thus, our data may reinforce the initial observation that T cell cytotoxicity was impaired in severe COVID-19 individuals [12]. Therefore, as Q-VD prevents CD8 T cell apoptosis, it could be a valuable tool for COVID-19 patients, not just by rescuing CD4 T cells but also by directly or indirectly improving the effector CD8 T cells. Interestingly, it has been reported that asymptomatic individuals developed a robust memory T cell response independently from humoral immunity [66, 67]. However, the recent demonstration that blocking Th1 cytokines reduces the pathogenesis of COVID-19 in mice raises the necessity for additional exploration into the role of T cells in COVID-19 [37]. Our current study focused on circulating blood T cells and therefore, further analyses assessing the occurrence of T cell apoptosis in lymphoid tissues would be of interest as it has been shown that T cells in the lymph nodes of COVID-19 patients are depleted [13].

Finally, our results highlighted higher levels of plasma sFasL correlating with CXCL10, a marker of disease severity [2, 5, 6]. Although activated T cells may produce FasL, it has also been shown that, after inflammation, non-lymphoid tissues may express FasL leading to T cell depletion [68]. In human fibrosis lung diseases, FasL are upregulated and associated with apoptosis of bronchiolar and alveolar epithelial cells [69, 70]. Furthermore, FasL can be released as a biologically active death-inducing mediator capable of inducing apoptosis during acute lung injury [71], and that metallopeptidases (including MMP7 as well as ADAM10) are responsible for FasL cleavage [72, 73]. This increase in both sFasL and CXCL10 could be related to p63 transcription [74]. Indeed, in the context of chronic obstructive pulmonary disease, the epigenetic reprogramming of p63 has been shown to be associated with a constitutive expression of transcripts such as CXCL10 [75]. Therefore, the question of whether infection of endothelial cells by SARS-CoV-2 induces the epigenetic reprogramming of p63 activation in pulmonary tissues, leading to CXCL10 expression and FasL expression, deserves to be further addressed. Furthermore, IL-18, which increases in COVID-19 patients, has been described to potentiate CXCL10 expression [76, 77]; a chemokine reported to inhibit cell proliferation [78–81] and promote the death of endothelial cells [82–84]; this process being prevented by administrating a caspase inhibitor [85]. Therefore, our observation regarding the relationship between plasma sFasL and CXCL10 is consistent with the idea of using Q-VD as an adjunctive molecule, which may also have a beneficial effect on the lung damage observed in SARS-CoV-2.

To conclude, our results indicate that severe/fatal SARS-CoV-2 infection is associated with apoptotic T cell death, which can be prevented by a pan caspase inhibitor, Q-VD. Thus, a strategy aiming to prevent T cell apoptosis could be beneficial in preventing the lymphopenia associated with severe disease outcome in COVID-19 patients.

## Materials and methods

### Study design and participants. overview of enrollment

The bioclinical features of the patients recruited at Nîmes University Hospital (France), from April 9th to July 16th, 2020 are shown in Table 1. Forty-one PCR-positive SARS-CoV-2-infected individuals were enrolled. Eleven patients were in the Intensive Care Unit (ICU) for acute respiratory distress syndrome. Thirty patients were admitted to the Infectious Diseases Department (non-ICU) for symptoms of dyspnea and/or deterioration in their general condition. Fourteen age- and sex-matched healthy controls were used as negative controls (age range, 28–95 years). This study was approved by the Île-de-France Ethics Committee) and all patients had provided written informed consent. The trial was registered as Eudract/IDRCB 2020-A00875-34 and ClinicalTrials NCT04351711. Blood samples were collected at a single time point upon hospital entry and plasma supernatant obtained after blood centrifugation was frozen at −80 °C. Cells were used for phenotyping and cell death quantification. We also analyzed sera samples obtained longitudinally at different time points of hospitalization from a separate cohort of 18 adult patients admitted for treatment of laboratory-confirmed COVID-19 from April 7th to May 7th 2020 (designated ‘longitudinal’ cohort). Blood was collected from these patients every 72 h, from admission until discharge, according to Hospital de Braga (Portugal) protocol, which was approved by the Clinical Board and Ethics Committee (ref 69/2020). Patients who did not completely follow the protocol, who had evidence of any simultaneous bacterial infection or patients treated with tocilizumab were excluded from the sample (Table 2).

### Flow cytometry

Cells were stained with specific antibodies as indicated in Supplementary Table 1. Cocktails of antibodies were used to define T and B cell subsets. Samples were analyzed by flow cytometry (Attune NxT, ThermoFisher) and using FlowJo software (Tree Star, Inc.).

### Cell death monitoring

Blood cells (5 × 10^5^ cells per ml) were cultured at 37 °C for 12 h in RPMI 1640 supplemented with 10% FCS (PAA Laboratories, Inc), penicillin/streptomycin (50 U/mL, Life technologies), glutamine (2 mM, Life technologies), sodium pyruvate (1 mM, Life technologies) and HEPES buffer (10 mM, Life technologies) at 37 °C and 5% CO_2_. Cells were cultured in the absence of presence of recombinant human FasL (200 ng/ml, Enzolifesciences). T cells were activated with either CD3 mAb (0.5 µg/ml, Becton Dickinson) or Staphylococcal enterotoxin B (SEB) (1 µg/ml, Sigma) in the absence or presence of either Q-VD (10 µM, MedChemExpres) or recombinant human Fas-Fc (5 µg/ml, Enzolifesciences). Cell death was assessed by measuring caspase-1 activity with FAM-FLICA caspase-1, caspase-3 activity with FAM-FLICA caspase-3 (Bio-Rad), active caspase-3 conjugated antibodies (R&D systems), and measuring phosphatidylserine (PS) exposure using labeled annexin V (Beckman Coulter Coultronics) according to the manufacturer’s instructions. Cells were stained with specific antibodies (supplementary Table 1). Samples were analyzed by flow cytometry (Attune NxT, ThermoFisher) and using FlowJo software (Tree Star, Inc.). Furthermore, cells were incubated in the absence or presence of several inhibitors, including IDN-6556 (10 µM, MedChemExpress), Q-VD-OPH (10 µM, MedChemExpress), VX-765 (10 µM, MedChemExpress), a NLPR3 inhibitor, MCC950 (1 µM, Merck) as well as necroptosis inhibitors GSK-872 and Dafrafenib (2 µM, MedChemExpress). The efficacy of these compounds was tested either in primary human cells or THP1 cell lines. Briefly, peripheral blood mononuclear cells (PBMC, 10^6^ cells per ml) were stimulated with 1 μg/ml of LPS (Sigma) for 24 h in the absence or presence of inhibitors. ELISA was used to quantify IL-1β (R&D Systems) in the supernatants of stimulated PBMC [53, 54]. THP1 monocytic cells were primed with LPS (1 μg/lml) for 6 h and then treated with ATP for 2 h (40 mM, Sigma). THP1 cells were also cultured with actinomycin D (ActD, Sigma) a conventional pro-apoptotic stimulus [52]. Jurkat cells were incubated in the presence of recombinant human FasL (Enzolifesciences) [51]. PS exposure was measured by flow cytometry.

### Quantification of cytokines and growth factors

ELISA (enzyme-linked immunosorbent assay) was performed to quantify the amounts of IL-18, FasL, sCD14, CXCL13, IL-1Ra, Caspase-1, TRAIL and IL-6 (Supplementary Table 2) in plasma from patients. An LDH detection Kit (Roche) was used and standard LDH quantification (Sigma) was prepared in two-fold serial dilutions starting from 0.3 U/ml. Plates were read at a reference wavelength of 490 nM. In parallel, human proinflammatory chemokines and growth factors were performed using LEGENDplex (Biolegend) according to the manufacturer’s instructions. Customized TruCulture® tubes (Myriad RBM) were purchased pre-filled with a proprietary medium along with anti-CD3 and anti-CD28 antibodies and a control (null). One ml of whole blood was collected directly in the tubes and then incubated at 37 °C. After 48 h, cells and supernatants were separated as recommended by the manufacturer and supernatants were stored at −80 °C.

### T cell purification and mRNA expression

CD4 and CD8 T cells were purified using anti-CD4 and anti-CD8 microbeads (Milenyibiotec) and lysed with TRIzol reagent (Thermo Fischer scientific). The mRNAs were extracted using a kit from Qiagen. RT-PCR was performed with 150 ng of RNA by a SensiFAST cDNA synthesis kit. Gene expression was assessed by qPCR using SensiFAST SYBR Hi-ROX kit (Bioline) in 10 µL reactions with 2.5 ng of cDNA. Thermocycling settings consisted of one hold for 15 min at 95 °C followed by a two-step temperature (95 °C for 15 s and 60 °C for 30 s) over 40 cycles in CFX384 Touch Real-Time PCR Detection System (Bio-Rad). Human-specific primers are described in supplementary Table 3 [86]. The transcript levels were normalized by the expression of the host housekeeping genes (*GAPDH* and *RPS18*). Given the low amount of primary T cells, each experiment represented a pool of three COVID-19 individuals (*n* = 12). Similar experiments were performed from healthy donors (three pools were used to define the baseline and calculate fold increase).

### Statistical analyses

Statistics were calculated using GraphPad Prism software. The non-parametric Mann–Whitney test was used for comparison. Correlations were assessed using the Spearman test. *P* values indicate significant differences (*, <0.05; **, <0.01; ***, *p* < 0.001; ****, <0.0001). A chi-squared test (_X_2 test) was used to compare frequency.

## Supplementary information

Supplemental figures and Tables

## Data Availability

The datasets used during the current study are available from the corresponding author on reasonable request. All data generated or analyzed during this study are included in this published article and its supplementary information files.
