# Supplementary material for: T cell apoptosis characterizes severe Covid-19 disease
Source: Cell Death Differ. 2022 Jan 22;29(8):1486–99. doi: 10.1038/s41418-022-00936-x (PMC8782710; doi:10.1038/s41418-022-00936-x)
Supplement: Supplementary file 1 — Supplemental figures and Tables [file 41418_2022_936_MOESM1_ESM.pdf]

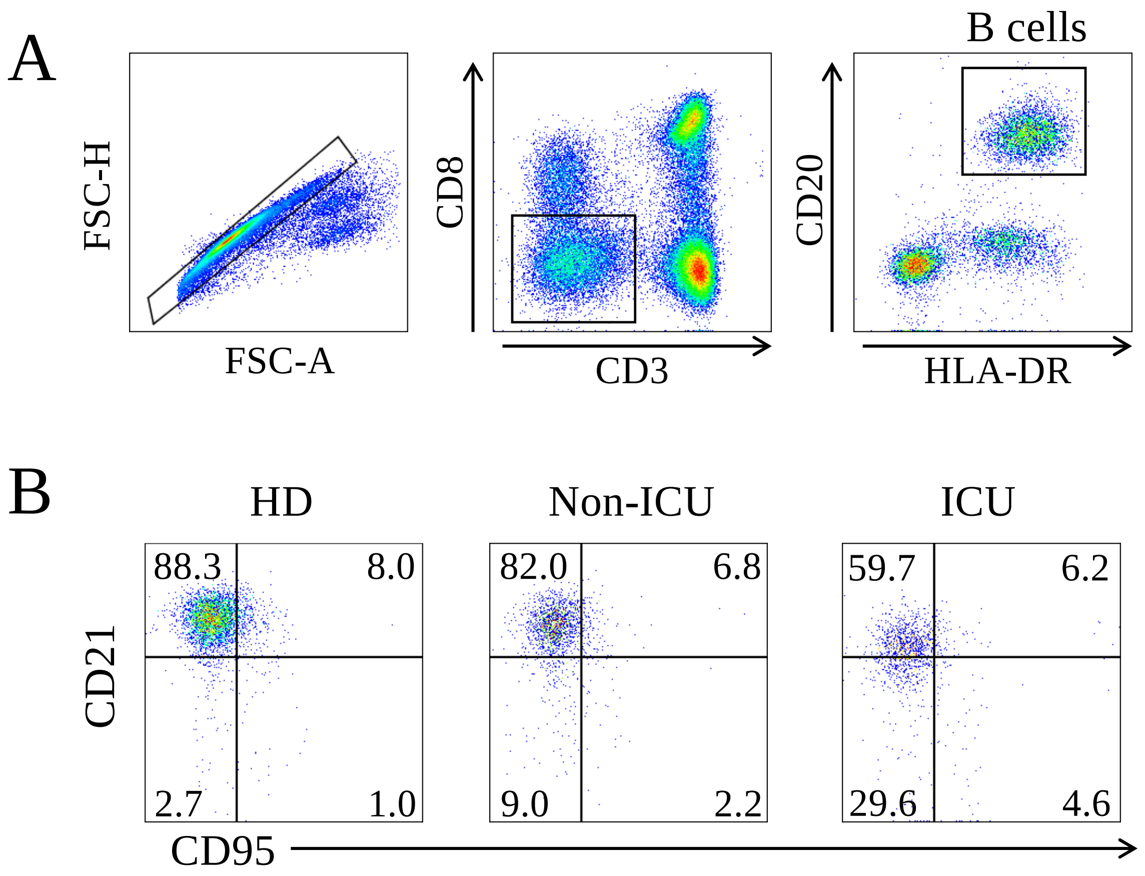

Supplementary Figure 1: CD95 expression on B cells. A) Gating strategy. B cells are defined by CD20+HLA-DR+ expression gating from CD3negCD8neg cells. B) Dot-plots showed CD95 expression on CD21+ B cells in HD, non-ICU and ICU individuals.

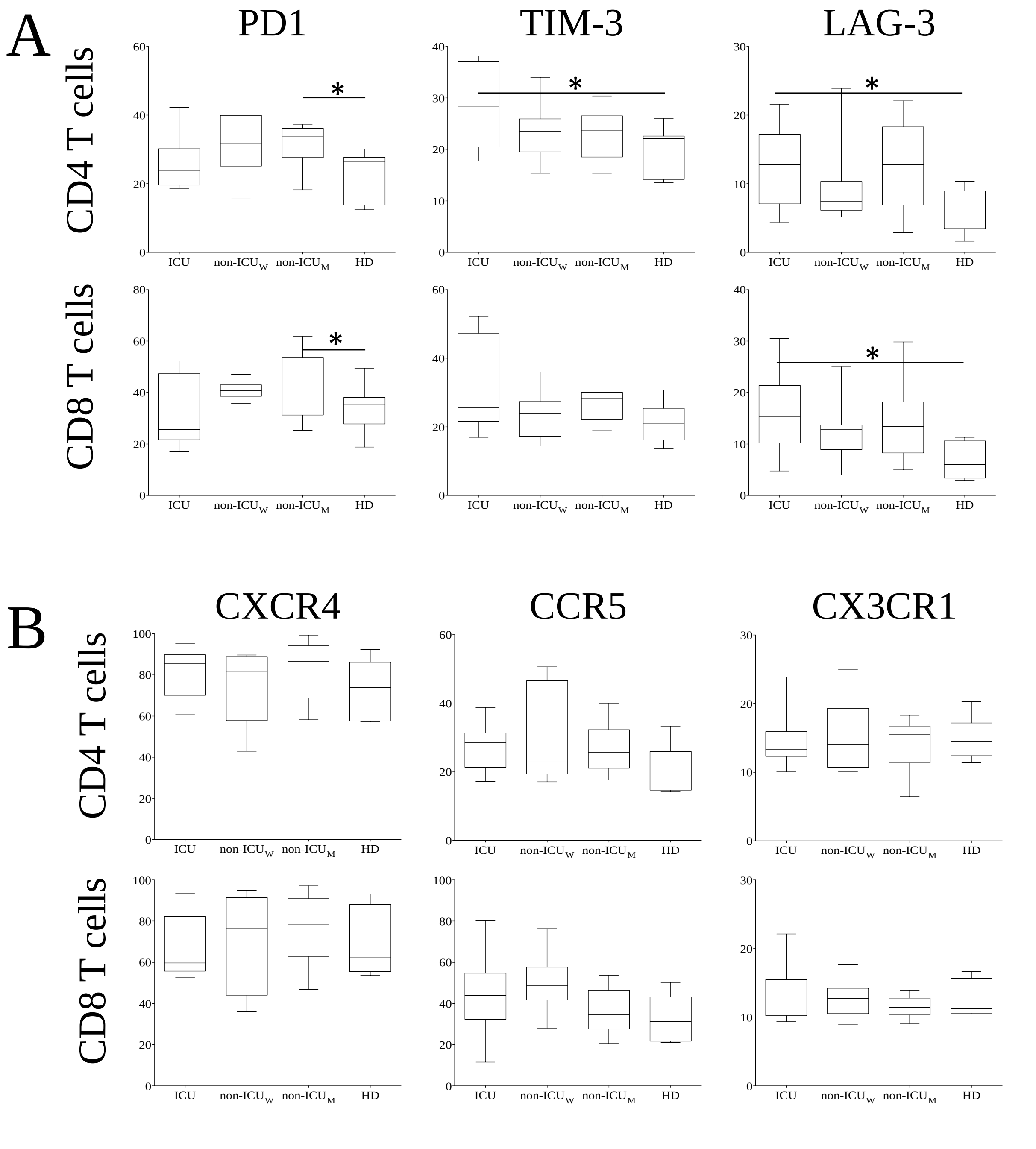

Supplementary Figure 2. Expression of exhaustion markers and chemokine receptors. A) Percentages of CD4 and CD8 T cells expressing exhaustion markers, PD1, TIM-3 and LAG-3, in ICU, non-ICU$_W$ (women) and non-ICU$_M$ (men) patients and HDs. B) Percentages of CD4 and CD8 T cells expressing chemokine receptors, CXCR4, CCR5 and CX3CR1. Statistical analysis was performed using a Mann-Whitney U test. *$p<0.05$.

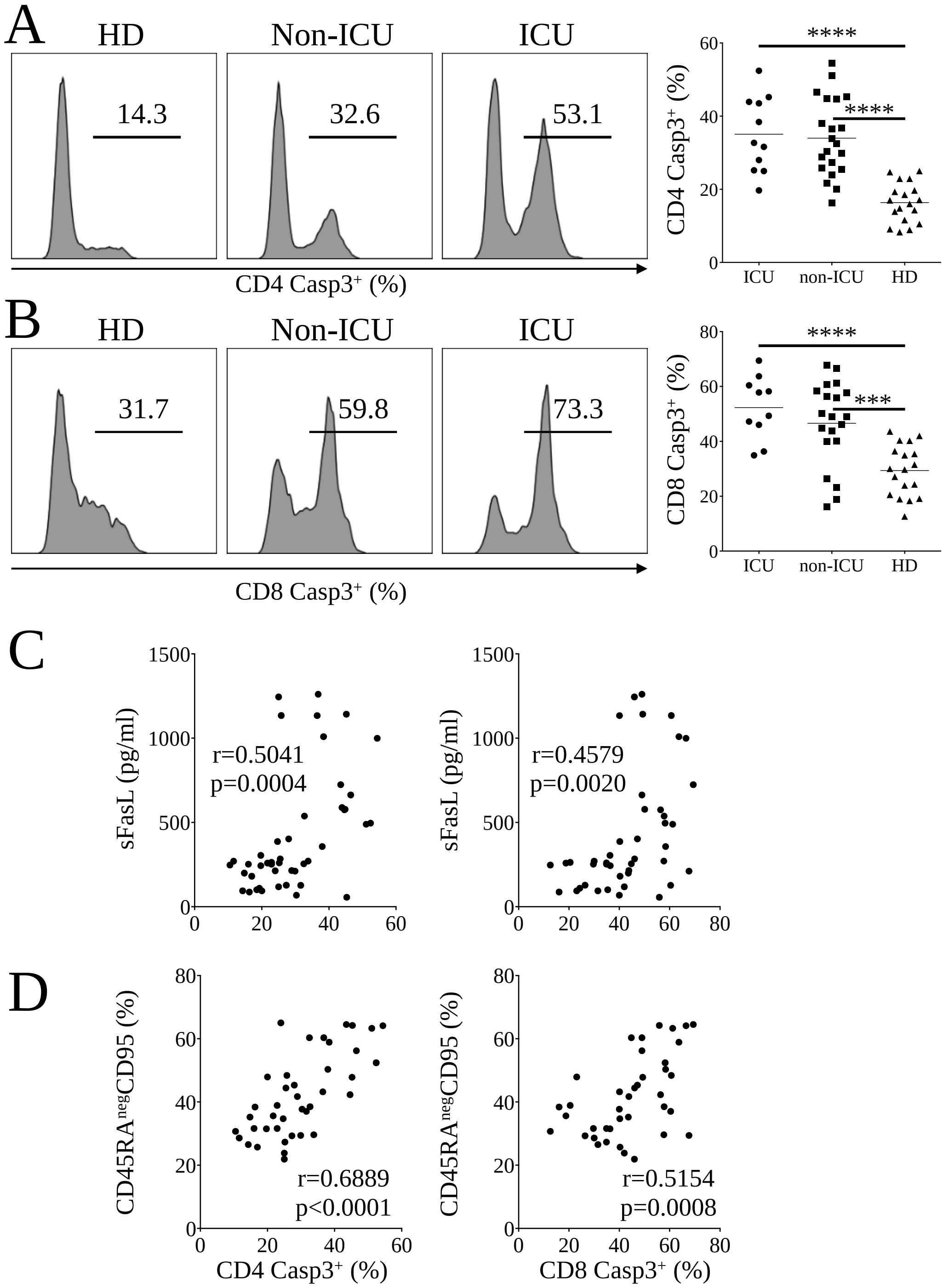

Supplementary Figure 3: Caspase-3 activation in T cells correlates with plasma sFasL and CD95 in COVID 19 patients. (A and B) Caspase activity of CD4 and CD8 T cells was quantified by flow cytometry using fluorescent caspase-3 substrate. Percentages of CD4 (A) and CD8 T cells (B) expressing fluorescent caspase substrates are shown. Each dot represents one individual. Statistical analysis was performed using a Mann-Whitney *U* test. ***p<0.001 and ****p<0.0001). C) Correlation between plasma sFasL and caspase-3 activation in CD4 and CD8 T cells. D) Correlation between the percentages of CD45RA$^-$ T cells expressing CD95 and caspase-3 activation in CD4 and CD8 T cells. Values of Spearman correlation are shown in the panels.

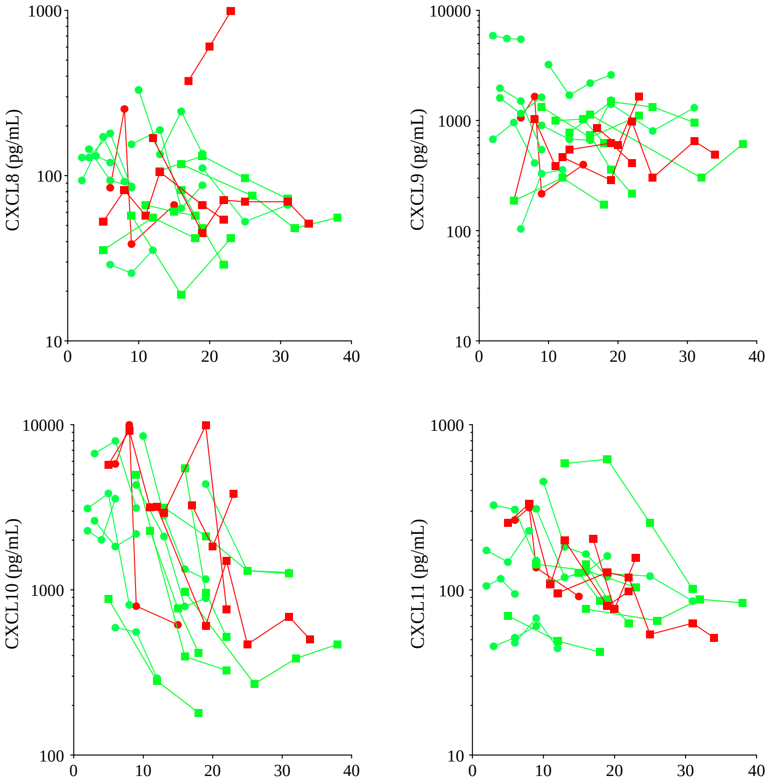

Supplementary Figure 4. Dynamics of cytokines in the sera of COVID-19. Cytokines were quantified in patients with severe infection (ICU, red) and with middle severity (non-ICU). Samplings were performed at different timepoints post symptomatology. Nine women and nine men were followed along the course of the disease.

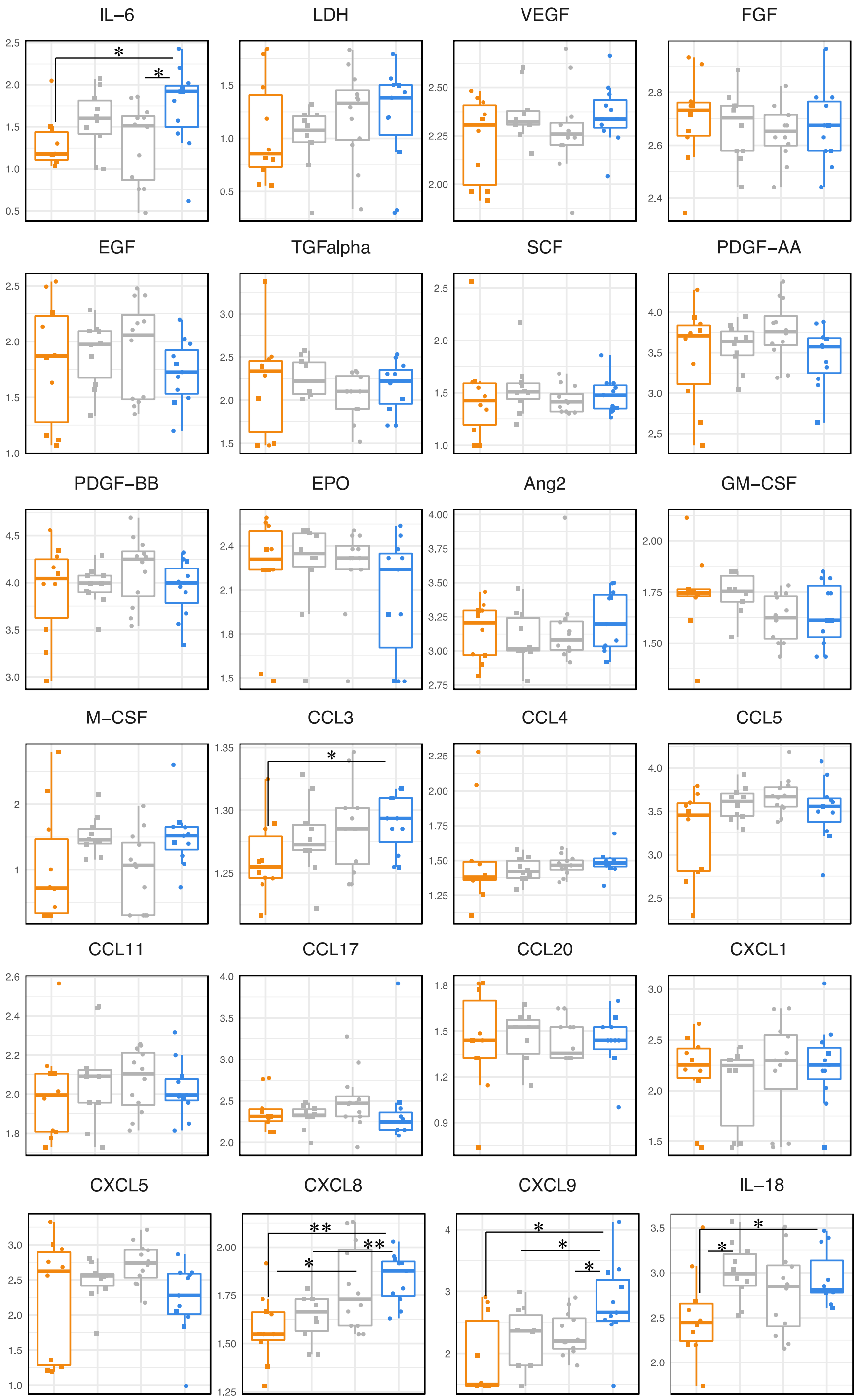

Supplementary Figure 5. Boxplots of the LFQ intensity values of cytokines for each patient. y-axis represents cytokine concentrations (pg/mL). Color represents the patient groups (orange: HD; gray: non-ICU; blue: ICU) and marker shapes represent sex (squared: men Boxplot2; rounded: women Boxplot3). Significant variations between groups based on a Student's t-test are showed above each graph (** $p<0.05$; *** $p<0.01$).

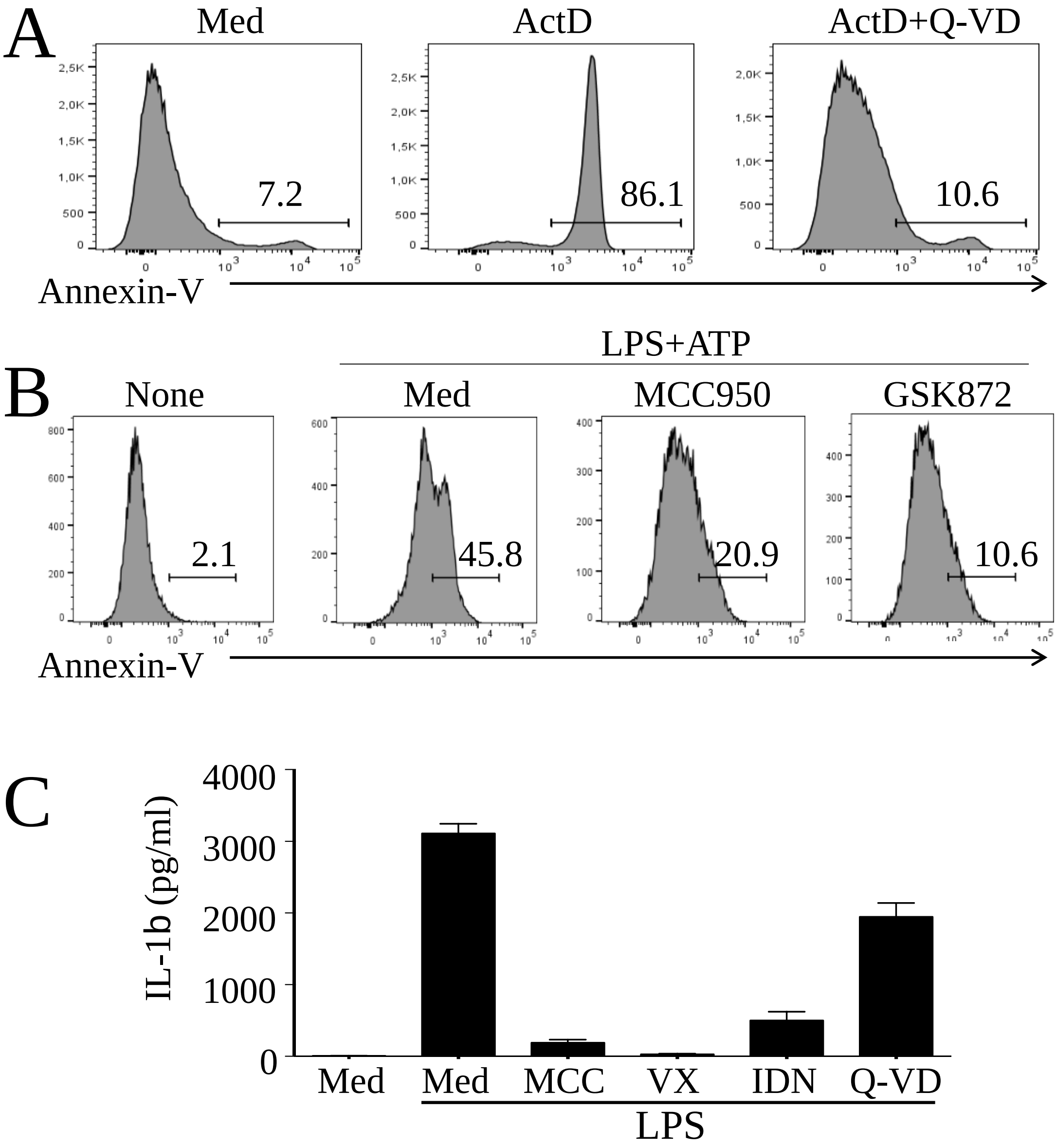

Supplementary Figure 6. A) THP-1 cells are treated for 8 hrs with actinomycin D (ActD, 2.5 µM) in the absence or presence of Q-VD (10 µM). Cell death was scored with annexin V-FITC staining by flow cytometry. Results are representative of at least two independent experiments. B) For necroptosis, THP-1 cells are primed with LPS (1 µg/ml) for 6 hr and then treated 2 hrs with ATP (40 µM). Cells were incubated in the absence or presence of either MCC950 (2 µM) or GSK872 (2 µM). Samples were scored with annexin V-FITC by flow cytometry. Data are representative of at least three independent experiments performed. C) PBMC are either unstimulated (Med) or stimulated overnight with LPS (1 µg/ml) in the absence or presence of MCC950 (1 µM), VX-765 (10 µM), IDN-6556 (10 µM), and Q-VD (10 µM). Supernatants are collected and IL-1β quantified by ELISA. Histogram is the mean of four healthy donors.

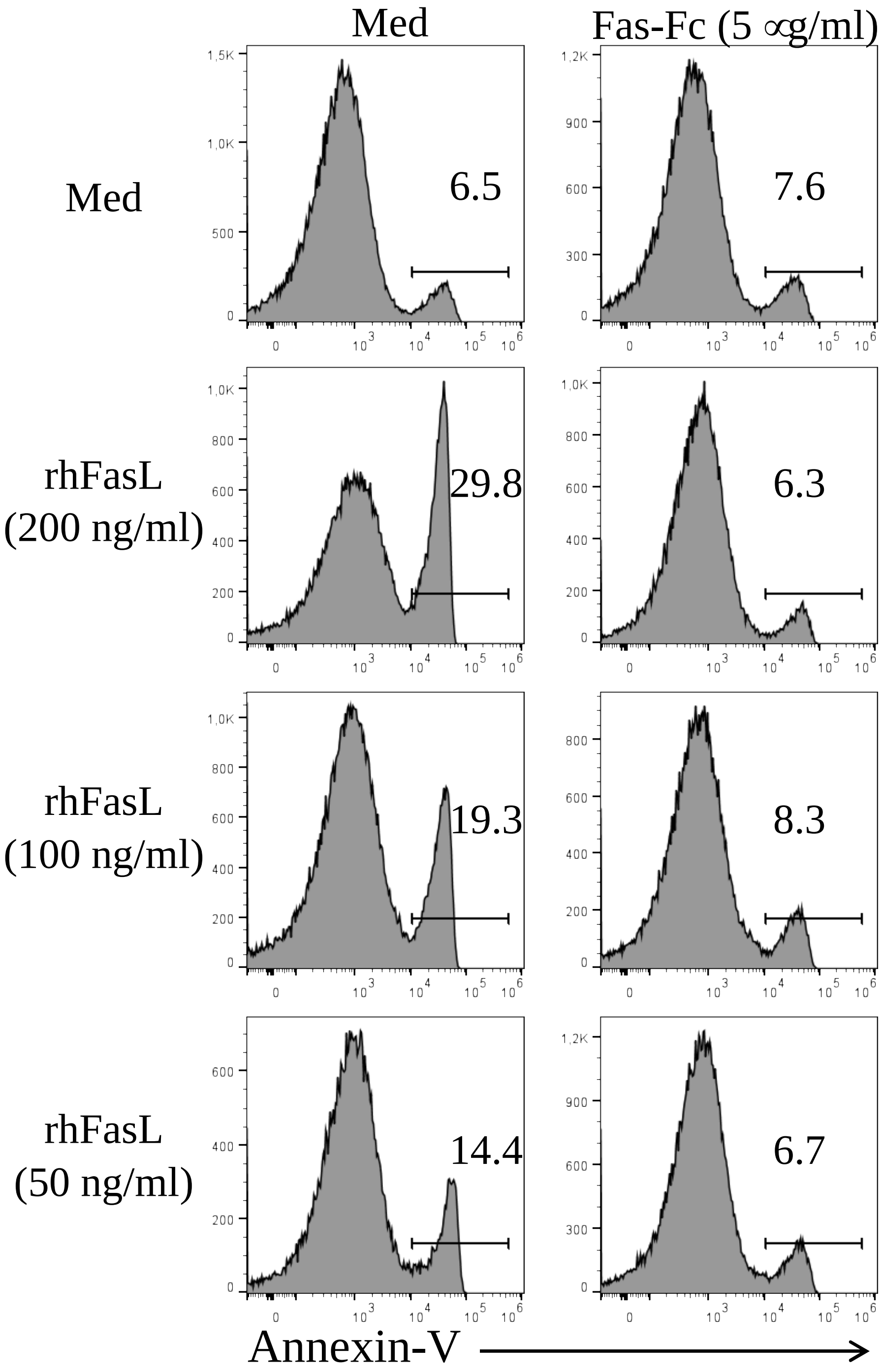

Supplementary Figure 7. Jurkat cells are treated for 8 hrs with different doses of recombinant human FasL (rhFasL) in the absence or presence of Fas-Fc (5 μg/ml). Cell death is scored with annexin V-FITC staining by flow cytometry. Results are representative of at least two independent experiments.

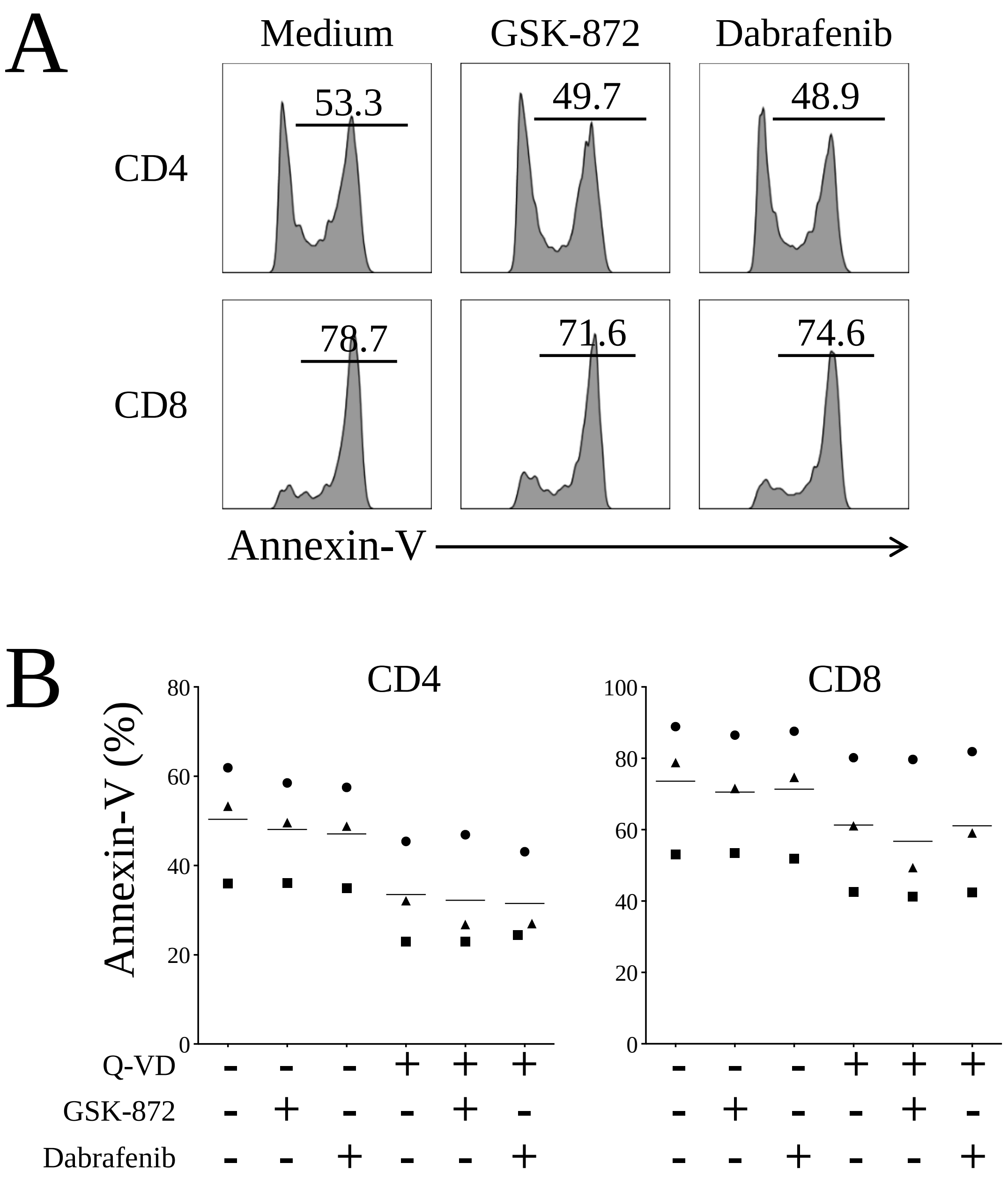

Supplementary Figure 8: RIPK3 inhibitors and T cell death. (A) Flow cytometry of CD4 and CD8 T cells from COVID-19 patients. (B) Percentages of CD4 and CD8 T cells expressing annexin-V are shown. Each symbol represents one individual. Cells were cultured in the absence of presence of Q-VD (10 µM) and incubated with or without GSK-872 (2 µM) and Dafrafenib (2 µM).

Antibody	Clone	Brand
CD3-vioblue	REA 613	Miltenyi Biotec
CD3-V500	SP34-2	BD Biosciences
CD3-PE-Cy7	SP34-2	BD Biosciences
CD8-PE	RPA-T8	BD Biosciences
CD8-PE-Cy7	RPA-T8	BD Biosciences
CD8-AF700	SK1	Biolegend
CD20-PE	2H7	BD Biosciences
CD20-PE-Cy7	2H7	BD Biosciences
CD20-APC-Vio770	REA 780	Miltenyi Biotec
CD20-APCH7	2H7	BD Biosciences
CD95-APC	DX2	Miltenyi Biotec
HLA-DR-PerCP-Vio700	REA 805	Miltenyi Biotec
CD45RA-PE-Vio770	REA 562	Miltenyi Biotec
CD4-ef450	OKT4	eBiosciences

Supplementary Table 1: Antibodies used.

Cytokine	Kit, Brand
IL-18	Human Total IL-18, R&D System
FasL	Human Fas Ligand/TNFSF6, R&D System
sCD14	Human sCD14, R&D System
CXCL13	Human CXCL13/BLC/BCA-1, R&D System
IL-1Ra	Human IL-1ra/IL-1F3, R&D System
TRAIL	Human TRAIL/TNFSF10, R&D System
IL-6	Human IL-6, R&D System
CXCL8, CXCL10, CCL11, CCL17, CCL2, CCL5, CCL3, CXCL9, CXCL5, CCL20, CXCL1, CXCL11, CCL4	Human Proinflammatory chemokine panel, Biolegend
Angiopoietin-2, EGF, EPO, FGF, G-CSF, GM-CSF, HGF, M-CSF, PDGF-AA, PDGF-BB, SCF, TGF-α, VEGF	Human growth factor panel, Biolegend

Supplementary Table 2: Cytokines, chemokines and factors tested in this study

TNF-α	FP : AGGCAGTCAGATCATCTTCTC<br>RP : GTTTGCTACAACATGGGCTAC
IFN-γ	FP : TTGGAAAGAGGAGAGTGACAG<br>RP : CATGTCTTCCTTGATGGTCTC
BAK	FP : CGCTGGGGAGACTGATAACT<br>RP : TGGCCTCAGGTAGAATGGTG
BAX	FP : GAGCTGCAGAGGATGATTGC<br>RP : TGGCAAAGTAGAAAAGGGCG
BCL-2	FP : ACTGGTGGAGGATGGAAAGG<br>RP : CCCAGGGCAGATTTTCCAAA
RIPK1	FP : TGGGCGTCATCATAGAGGAAG<br>RP : CGCCTTTTCCATGTAAGTAGCA
RIPK3	FP : AATTCGTGCTGCGCCTAGAAG<br>RP : TCGTGCAGGTAAAACATCCCA
MLKL	FP : AGGAGGCTAATGGGGAGATAGA<br>RP : TGGCTTGCTGTTAGAAACCTG
RPS18	FP : CTGCCATTAAGGGTGTGG<br>RP : TCAATGTCTGCTTTCCTCAAC
GAPDH	FP : CAACTACATGGTTTACATGTTCC<br>RP : GAAGATGGTGATGGGATTTC

Supplementary Table 3: primers used to quantify mRNA transcripts
